# Holistic processing is modulated by the probability that parts contain task-congruent information

**DOI:** 10.3758/s13414-023-02738-w

**Published:** 2023-06-13

**Authors:** Kim M. Curby, Lina Teichmann, Mary A. Peterson, Sarah S. Shomstein

**Affiliations:** 1 School of Psychological Sciences and the Performance and Expertise Research Centre, Macquarie University, Sydney, NSW Australia; 2 Laboratory of Brain and Cognition, National Institute of Mental Health, National Institutes of Health, Bethesda, MD USA; 3 Cognitive Science Program and Department of Psychology, University of Arizona, Tucson, AZ USA; 4 Department of Psychology, George Washington University, Washington, DC USA

**Keywords:** Holistic processing, Face processing, Probability, Attention

## Abstract

Holistic processing of face and non-face stimuli has been framed as a perceptual strategy, with classic hallmarks of holistic processing, such as the composite effect, reflecting a failure of selective attention, which is a consequence of this strategy. Further, evidence that holistic processing is impacted by training different patterns of attentional prioritization suggest that it may be a result of learned attention to the whole, which renders it difficult to attend to only part of a stimulus. If so, holistic processing should be modulated by the same factors that shape attentional selection, such as the probability that distracting or task-relevant information will be present. In contrast, other accounts suggest that it is the match to an internal face template that triggers specialized holistic processing mechanisms. Here we probed these accounts by manipulating the probability, across different testing sessions, that the task-irrelevant face part in the composite face task will contain task-congruent or -incongruent information. Attentional accounts of holistic processing predict that when the probability that the task-irrelevant part contains congruent information is low (25%), holistic processing should be attenuated compared to when this probability is high (75%). In contrast, template-based accounts of holistic face processing predict that it will be unaffected by manipulation given the integrity of the faces remains intact. Experiment 1 found evidence consistent with attentional accounts of holistic face processing and Experiment 2 extends these findings to holistic processing of non-face stimuli. These findings are broadly consistent with learned attention accounts of holistic processing.

## Introduction

An abundance of research has established the holistic nature of the processing style that supports skilled perception of faces and other objects of expertise. Holistic processing, that is, the processing of a stimulus as a unified whole, rather than as a collection of features, is a key focus of research aimed at understanding face perception. However, there is still much unknown about what mechanisms underlie this processing strategy. There is growing evidence that it may be supported, at least in part, by shaping, through learning, how these objects are attended (Chua et al., 2014). One possibility is that, because the information required to identify faces accurately and efficiently is spatially distributed across the face, a holistic attentional strategy is optimal and thus develops through experience processing these stimuli. This may be especially the case because the typically homogenous nature of face stimuli may render a simpler, more spatially constrained, strategy inadequate. If so, it is possible that if adopting a distributed attentional strategy was no longer optimal for the task at hand, participants may process the faces less holistically. Here we address this possibility by comparing holistic processing as measured in the composite face paradigm under conditions where there is a high, compared to a low, probability that the task-irrelevant face region will contain misleading information, that is, incongruent with the correct judgement about the task-relevant part. Notably, this manipulation specifically sets up contrasting conditions where a holistic attentional strategy is either detrimental or advantageous for performance.

Holistic processing is one of the most robust features distinguishing the processing of faces and non-face objects of expertise from that of other objects (e.g., Curby & Gauthier, 2014; Gauthier & Tarr, 2002). The composite face task is commonly used to tap and demonstrate this processing style as it reveals a characteristic feature of holistic processing, that is, the apparent obligation to process stimuli as wholes, irrespective of the task at hand. In this task, when making a judgement about one half of the face, participants’ experience interference from the other, task-irrelevant half (Young et al., 1987). The degree to which manipulations of the task-irrelevant part impact judgements involving only the task-relevant part provides an index of holistic perception. A core finding in this task is that part-matching judgements about composite faces are influenced by the compatibility of the task-irrelevant face parts with the correct judgement about the task-relevant parts (i.e., whether it would require the same [congruent] or a different [incongruent] response as that for the task-relevant part; *congruency effect*) (e.g., Curby et al., 2013). Performance is better when the relationship between the task-irrelevant parts (i.e., whether they are the same or different) is the same as that between the task-relevant parts. Notably, this impact of the task-irrelevant part, i.e., the congruency effect, implies that participants are processing both parts of the face, and is taken to imply that the face is being processed holistically.

Some accounts of holistic face processing have traditionally highlighted and emphasized the importance of the prototypical configuration of features within faces. According to this account, the prototypical facial configuration is critical in triggering or allowing access to face-specific mechanisms that encode the face as a singular unit relative to a face template (Farah et al., 1998; Morton & Johnson, 1991). More specifically, it has been proposed that holistic processing is the result of an early, obligatory detection stage that relies on a coarse template of an upright face (Tsao & Livingstone, 2008). Some even suggest that holistic processing occurs before face detection, serving to support face detection (Taubert et al., 2011) and operating in the absence of attention (Boutet et al., 2002; Lavie et al., 2003). Notably, this account explains the composite effect as a consequence of the aligned face parts being detected as a singular face unit and therefore encoded and processed as a unit (Tsao & Livingstone, 2008). Support for the importance of facial configuration in triggering these early processes comes from studies demonstrating that disrupting the configuration of facial features, via a variety of different manipulations such as inversion, or scrambling or misaligning the parts, impairs holistic processing (e.g., Tanaka & Farah, 1993; Tanaka & Sengco, 1997; Yin, 1969; Young et al., 1987).

In contrast to accounts that propose that holistic processing is triggered in an obligatory fashion by stimulus-based factors, specifically the match with an internal face template, more recently holistic face processing has been re-framed as an attentional strategy. According to this account, somewhat inflexible attentional weightings to features within faces and other objects of expertise are developed through experience with these stimuli. In the context of the typical composite task where participants are asked to make a judgement about only a part (top or bottom) of a stimulus, the inflexibility of these attentional weightings results in difficulty attending to only the task-relevant part. This results in what appears to be a failure of selective attention. This learned attentional account for holistic processing can be similarly applied to other measures of holistic processing where participants appear obliged to process the whole face stimulus despite being asked to make a judgement about only a part, such as the part-whole effect (Tanaka & Farah, 1993), or when the stimulus is presented in a novel manner (e.g., inverted) and thus is less constrained by attentional weightings more strongly tied to the familiar (upright) orientations. These weightings presumably reflect the task-relevance, based on the observer’s experience, of the different information with the face or object. Consistent with this account, a recent series of studies has found that patterns of behaviour that have been defined as hallmarks of holistic processing can be eliminated or strengthened by training in different patterns of attentional prioritization (e.g., Chua et al., 2014; Chua et al., 2015). For example, if a particular part of a trained stimulus is unlikely to be diagnostic, i.e., it rarely contains task-relevant information, participants learn to not attend to this part (i.e., to prioritise other parts of the stimulus). In this case, holistic perception does not occur, and participants are better able to ignore the non-diagnostic (task-irrelevant) part (Chua et al., 2014, 2015). However, these studies used unnatural stimuli, either novel race faces or novel objects, namely Greebles.

Notably, the manipulations used in previous studies demonstrating that holistic processing can be shaped by learned attentional strategies were tied to specific parts or spatial regions of stimuli. Specifically, the region of the stimulus that failed to contain diagnostic information was fixed for participants, that is, it was always either the top *or* the bottom across the participant’s experience with stimuli from that category. Notably, the shaping of attention in these studies could be explained by learned attention to, or a prioritisation of, specific regions within the different stimulus sets. An open question is whether such an impact of learned attention would still occur if the location of the diagnostic information were not spatially fixed, but rather a holistic attentional strategy was discouraged or encouraged by the probability that the task-irrelevant part, whichever it may be, will contain misleading or consistent information. If so, manipulating the probability that the task-irrelevant region of the faces in the composite part-matching task contain congruent or incongruent information should impact the degree to which they are processed holistically. This finding would argue against a more simplistic spatial attention account of holistic processing.

Participants, by default, process the face stimuli presented in composite face tasks holistically, despite the task requiring them to make a same/different judgement about only a part of the faces. However, based on feedback about the accuracy of their judgements, participants may be able to learn to process the faces in such a way as to minimise the interference from the task-irrelevant part. Specifically, participants could learn, via incidental, statistical learning, the probability that the task-irrelevant part or region will contain congruent (consistent) or incongruent (misleading) information. If the task-irrelevant region has a high probability of containing misleading information, that is, information that is inconsistent with accurate performance, then participants might be able to change the way they process the faces, minimising the degree to which the task-irrelevant part is processed. In contrast, if holistic processing of faces is driven by an obligatory template-based encoding process, then the probability that task-irrelevant parts of the face contain congruent or incongruent information (i.e., the task context) should have no effect on holistic processing as this should be similarly triggered by the face stimuli irrespective of the probability that the task irrelevant part will contain incongruent (or congruent) information.

## Experiment 1

Here we probe the attentional account of holistic processing by examining if manipulating the probability that the task-irrelevant face part in the composite face task will contain task-congruent or task-incongruent information impacts the degree to which people process natural faces holistically. An attentional account of holistic processing would predict that when the probability that the task-irrelevant part contains congruent information is low, holistic processing should be attenuated compared to when this probability is high. Specifically, when there is a low probability (25%) that the relationship between the task-irrelevant parts is congruent with that between the task-relevant parts (i.e., both are the same or both are different), the congruency effect should be reduced compared to when this probability is high (75%). In contrast, a template-based account of holistic processing of faces would predict that holistic processing will be unaffected by the probability that the task-irrelevant face part will contain task-congruent or task-incongruent information.

### Method

#### Participants

Forty-seven undergraduate students (38 female, mean age = 21.8 years, SD = 5.66) were recruited to participate in this study for course credit. Sample size was preregistered and was determined based on a power analysis assuming a small-to-medium effect size and power of at least .80.^1^ All participants reported normal or corrected-to-normal vision and gave their informed consent before participating.

#### Stimuli

The main stimuli consisted of 12 greyscale front-view images of male faces wearing neutral expressions from the Karolinska Directed Emotional face (KDEF) database (Lundqvist et al., 1998). The images were cropped to remove the hair and ears and were cut in half to obtain a top and bottom half (each part was 4.4 × 3.2° of visual angle). Four additional face stimuli were used in the practice trials.

#### Apparatus and procedure

**Fig. 1:**
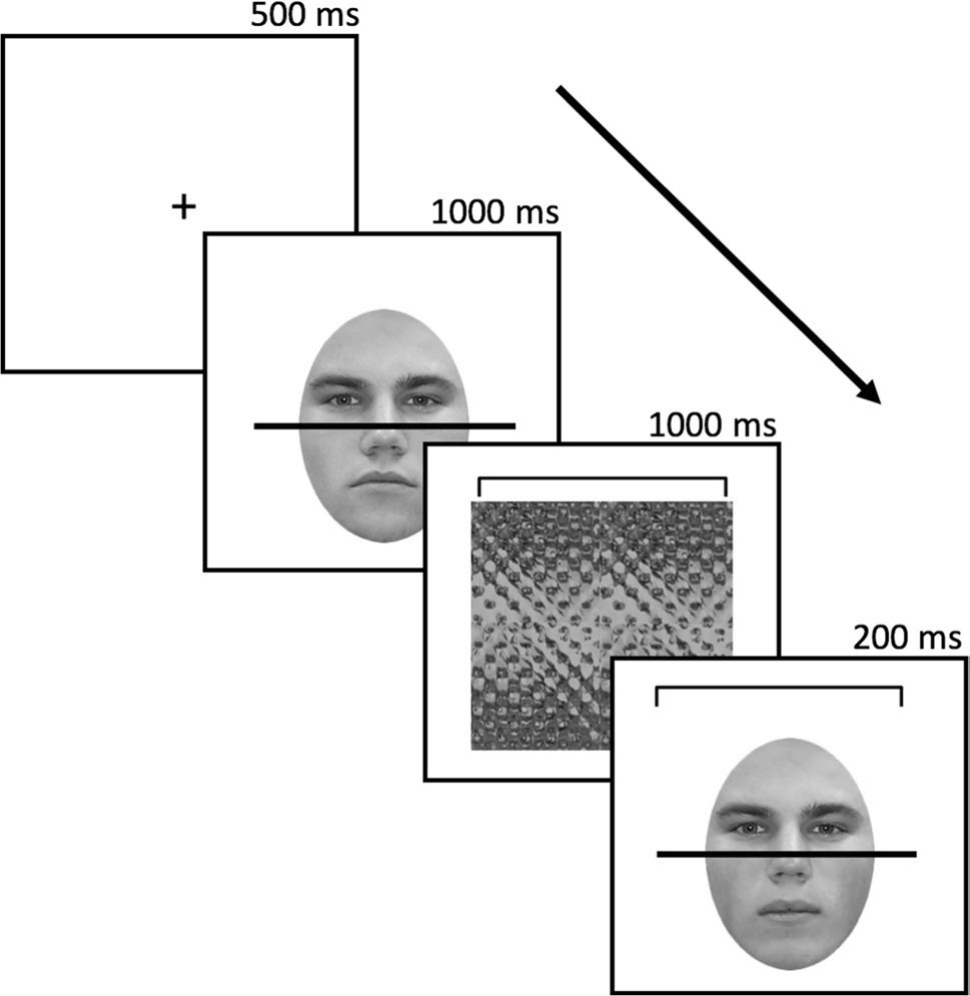
Trial structure used for the composite face task. A bracket served as the cue in each trial to indicate which part (top or bottom) the participant should make a same/different judgement on. In the example, the bracket is around the top half, indicating the matching judgement should be performed on the top part. Depending on the condition, the relationship between the task-irrelevant parts (the bottom parts here) was congruent or incongruent with that between the task-relevant parts (the tops parts here). In the example, the task-irrelevant parts are incongruent as they are different, while the tops parts are the same

The stimuli were viewed on a 24-in. monitor with a resolution of 1,920 × 1,080 pixels, at approximately 60 cm (head position was not controlled), in a dark room. The experiment used the Psychophysics Toolbox (PTB3) extensions of Matlab software (Brainard, 1997; Kleiner et al., 2007; Pelli, 1997). Participants were tested individually or in pairs. All participants performed a modified version of what is typically referred to as the complete version of the composite face task (see Fig. 1). This version of the task was chosen as it includes both congruent and incongruent conditions, which is critical for our manipulation, whereas the traditional version only includes the incongruent condition. As in the standard version of this task, face composites were created by combining the top of one face with the bottom of another face. A 0.05 dva horizontal black line was drawn in the middle in order to clearly separate the top and bottom halves.

Each trial started with a central fixation cross (500 ms), followed by a composite (chimeric) face comprising the top and bottom parts of different faces (1,000 ms). This was masked by a textured pattern (for 1,000 ms) that also contained a bracket around either the top or bottom part indicating which part was the task-relevant part. Following the mask, a second face and the bracket cue were briefly presented (200 ms). Participants’ indicated via a key press whether the cued half (top or bottom) of the second face was the same or different in identity to that of the first face. After entering their response via a key-press on a keyboard, participants were given feedback if they made an error (i.e., the text “Incorrect” appeared on the screen). Participants also received feedback if they failed to enter a response before the trial timed out (2,500 ms; the text “No Response” appeared on the screen). The feedback was shown for 500 ms and a fixation cross was presented for 1,000 ms after the feedback.

In some trials the same/different relationship between the task irrelevant (non-cued) face parts in the two faces was *congruent* with the relationship between the task-relevant (cued) parts. In other words, in congruent trials, if the task-relevant parts differed between the two faces, thus rendering the correct response for the trial “different”, the task-irrelevant face parts also differed. In others trials, the same/different relationship between the task irrelevant (non-cued) face parts in the two faces was *incongruent* with the relationship between the task-relevant (cued) parts. For example, if the task-relevant parts differed between the two faces, thus again rendering the correct response for the trial “different”, the task-irrelevant face parts were the same.

Before each experimental session, participants completed 32 practice trials. In the practice trials, the proportion of congruent and incongruent trials was equal. Participants were offered a break after every 64 trials**.** Participants completed six blocks of 64 trials for a total of 384 trials in each of the two sessions.^2^ Within each session either 288 of the trials were congruent and 96 were incongruent (high proportion congruent condition) or 96 of the trials were congruent and 288 were incongruent (low proportion congruent condition), depending on the condition. The order in which the two proportion conditions (sessions) were completed was counter-balanced across participants. The first two blocks in each session were used to establish the contextual probability manipulation. Sensitivity scores (d’) were calculated using the hit rate and false alarm rates for each condition and for each participant from the data from the remaining blocks in each session (blocks three through six).

After completing the study, some participants spontaneously commented on the one session (either the first or the second) being more difficult, but none of their comments suggested that they were aware of the difference between the two sessions of the study. After the first 16 participants, we started to ask participants an open-ended question about how they found the two sessions and how they compared, and we recorded any differences they noted between the two sessions.

### Results and discussion

**Fig. 2:**
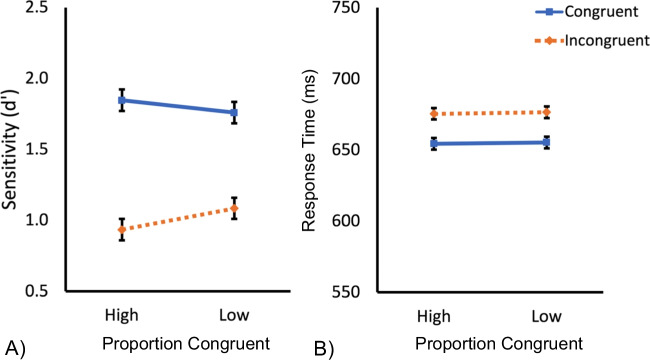
(**A**) Mean sensitivity (d′) for the congruent (solid blue) and incongruent (dashed orange) trials in the high and low proportion congruent conditions for the face stimuli in Experiment 1. (**B**) Mean response time (ms) for the congruent (solid blue) and incongruent (dashed orange) trials in the high and low proportion congruent conditions. Error bars represent standard error values

Data from two participants were excluded as they only completed one session of the experiment. Data from an additional two participants were excluded as they incorrectly completed the same session twice. Sensitivity scores (d’) were calculated using the hit rate and false alarm rates for each condition and for each participant. The data from one participant were excluded due to chance-level performance (mean d’ ~ 0). The mean response time (RT; ms) and sensitivity scores (d’) for each condition for the remaining participants are presented in Fig. 2.

#### Sensitivity (d’)

A 2 (congruency; congruent, incongruent) × 2 (proportion congruent; high [75% congruent/25% incongruent], low [25% congruent/75% incongruent]), repeated-measures ANOVA was performed on these data. The analysis revealed a main effect of congruency, *F*(1, 41) = 112.01, *p* ≤ .0001, η_p_^2^ = .73, with the expected higher sensitivity for congruent trials than incongruent trials, suggesting a failure to selectively attend to the task-relevant part of the faces. There was no main effect of proportion congruent, *F*(1, 41) = 0.17, *p* = .68, η_p_^2^ = .0042. However, there was a significant interaction between congruency and proportion congruent, *F*(1, 41) = 5.00, p = .031, η_p_^2^ = .11, with a larger effect of congruency in the high, relative to the low, proportion congruent condition (Fig. 2A.). The impact on the congruency effect, an index of holistic processing, of manipulating the probability that the task-relevant part will contain congruent information is consistent with learned attentional accounts of holistic processing: Participants benefit more from congruent trials and show more interference from incongruent trials in the high-congruency than the low-congruency condition.

#### Response time (RT)

The mean RT for each condition for correct trials only is presented in Fig. 2B. A 2 (congruency; congruent, incongruent) × 2 (proportion congruent; high [75% congruent/25% incongruent], low [25% congruent/75% incongruent]), repeated-measures ANOVA was performed on these data. The analysis revealed a main effect of congruency, *F*(1, 41) = 28.24, *p* ≤ .0001, η_p_^2^ = .41, with the expected faster RTs for congruent trials than incongruent trials. There was no main effect of proportion congruent, *F*(1, 41) = 0.004, *p* = .95, η_p_^2^ < .0001, or interaction between congruency and proportion congruent, *F*(1, 41) = 0.001, p = .97, η_p_^2^ < .0001 (see Fig. 2B.). These findings suggest that the effect of manipulating the probability that the task-irrelevant part will contain congruent or incongruent information is limited to sensitivity measures.

During the debriefing session after the completion of the study, participants were probed as to whether they noticed any differences between the sessions. Notably, participants were not directly asked whether they were aware that the task-irrelevant part had a higher or lower likelihood of being congruent or incongruent in the different sessions. Asking directly could have cued their attention to the manipulation post-study even if they were not aware of it while performing the task. No participants reported being aware of any difference related to the task-irrelevant part. Participants did frequently report finding the session in which they completed the low congruent condition, whether it was completed first or second, more difficult (20 out 30 participants asked reported that they found this condition more difficult). Notably, despite this, there was no main effect of congruency probability on sensitivity measures. Intriguingly, more than half of these participants explained away the difference in difficulty by reporting, for example, that they were more tired or that they had a headache during this session. For those who completed this session first, they generally attributed the reduced difficulty of the second session, that is, the high congruent condition, to benefits that they had accrued though practise during the first session. More than half (12 out of 20) explained their perceived difference in performance in this way. This pattern of responses is consistent with the suggestion that the modulation of the size of the congruency effect by the probability that the task-irrelevant face part would be congruent or incongruent occurred implicitly, that is without participants awareness. Future studies should further probe the nature of this effect, specifically the degree to which these effects occur implicitly.

While we cannot definitively report that participants were unaware of the congruency probability manipulation, there are several factors that would have made it difficult for participants to detect this difference between the two sessions. First, the fact that our manipulation only changed the task-irrelevant part, that is, the part that should be ignored based on the task instructions, would have made it less likely that participants would become aware of the manipulation. Second, the probabilistic, rather than absolute, nature of the manipulation would have also made it less likely to be noticed. Third, since it was a relationship between stimulus parts that was manipulated and not the simple presence or absence of a feature, this would have also made it harder to detect. Finally, the fact that the manipulation did not change the expected (correct) response, that is, 50% of the trials in both conditions required a “same” response and 50% required a “different” response, would have also made it more difficult to detect the difference between the two sessions. Thus, there are several factors that would have made it difficult for participants to notice and identify the difference between the sessions, reducing the likelihood that they were able to develop a distinct, explicit strategy for the different sessions.

Notably, the reduced impact of the task-irrelevant part on performance in the low-congruency, compared to the high-congruency, condition in Experiment 1 cannot be explained by participants adopting a simple attentional strategy whereby a specific stimulus part or region is suppressed (or prioritised). Across the study, both the top and bottom regions of the stimuli were equally likely to be the target (i.e., task-relevant) on any given trial, rendering a set space-, or even feature-, based suppression strategy ineffective. In addition, not only was the spatial location to be attended not predictable in the current study, but participants were only informed which region was task-relevant after the first stimulus on a trial was presented (and removed).

## Experiment 2

The findings of Experiment 1 are consistent with a learned attention account of holistic face processing. Our findings suggest that holistic processing of faces is, at least in part, shaped by the task context. This presumably occurs via participants’ ability to learn, through experience, to shape their attention so that it is better optimised for the task at hand. More recently, non-face objects rich in Gestalt cues have been shown to demonstrate the same markers of holistic processing that have been seen for faces, that is, a congruency effect and a disruption to the effect of congruency when the configuration of the Gestalt stimulus is disrupted by misalignment of parts within it (Zhao et al., 2016). Recent studies have also provided evidence that the holistic processing of these stimuli rich in Gestalt cues recruit partially overlapping mechanisms with those underlying holistic face processing (Curby et al., 2019; Curby & Moerel, 2019). Here we investigate whether the holistic processing of stimuli rich in Gestalt cues, like those used by Zhao et al. (2016), can be impacted by manipulations of the probability that the task-irrelevant part will contain congruent or incongruent information. If similar mechanisms underlie the holistic processing of these Gestalt stimuli as those for faces, a similar pattern of findings to those found in Experiment 1 should be present. Specifically, when the probability is low (25%) that the relationship between the task-irrelevant parts will be congruent with that between the task-relevant parts (i.e., both are the same or both are different), the congruency effect should be attenuated compared to when this probability is high (75%).

### Method

#### Participants

Forty-nine undergraduate students (34 female, mean age = 21.1 years, SD = 5.644) were recruited to participate in this study for course credit. Sample size was preregistered and was determined based on a power analysis assuming a small-medium effect size and power of at least .80.^3^ All participants reported normal or corrected-to-normal vision and gave their informed consent before participating.

#### Stimuli

**Fig. 3:**
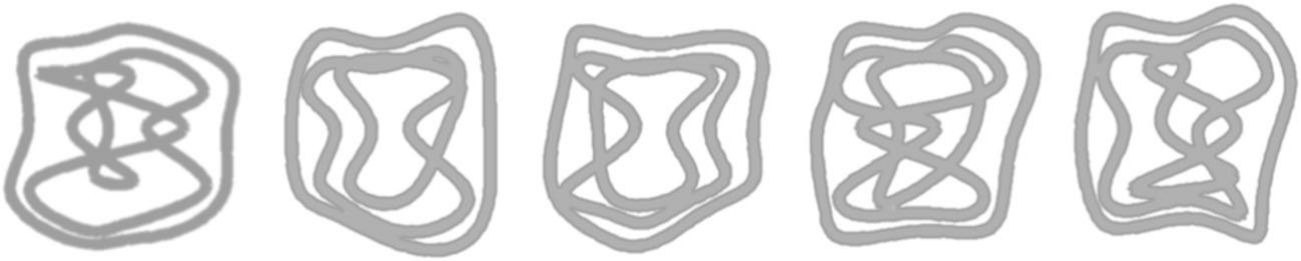
Examples of the Gestalt line pattern stimuli used to make the composite stimuli in Experiment 2. The lines within all the stimuli intersected the horizontal midline at the same six locations, allowing any top and bottom regions of the different images to be combined without disrupting the Gestalt cues within the stimuli

The main stimuli consisted of 12 line pattern drawings, modelled after those created by Zhao et al. (2016), that were generated using Matlab (see Fig. 3 for examples). Prior research has demonstrated that the processing of these stimuli, like the stimuli created by Zhao et al. (2016), show key hallmarks of face-like holistic processing revealed by composite tasks, that is, a congruency effect and an interaction between congruency and misalignment (Curby & Teichmann, 2022). The images were cut in half to obtain a top and bottom half (each part was 4.4 × 3.2° of visual angle). Notably, the line patterns were constrained so that all lines intersected the vertical halfway point of the images at the same four locations so that the top and bottom parts could be interchanged seamlessly across images. Four additional line pattern stimuli were used in the practice trials.

#### Apparatus and procedure

The apparatus and procedure were the same as in Experiment 1 with the only difference being the task was performed with line pattern stimuli and not faces.

### Results and discussion

**Fig. 4:**
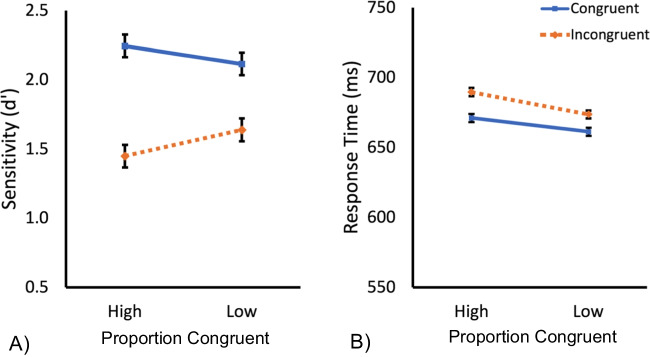
(**A**) Mean sensitivity (d′) for the congruent (solid blue) and incongruent (dashed orange) trials in the high and low proportion congruent conditions for the line stimuli in Experiment 2. (**B**) Mean response time (ms) for the congruent (solid blue) and incongruent (dashed orange) trials in the high and low proportion congruent conditions. Error bars represent standard error values

Data from three participants were excluded as they only completed one session of the experiment. Sensitivity scores (d’) were calculated using the hit rate and false alarm rates for each condition and for each participant. The data from three participants were excluded due to below or chance-level performance (mean d’~ 0 or lower). The mean sensitivity scores (d’) and RT (ms) for each condition for the remaining participants are presented in Fig. 4.

#### Sensitivity (d’)

A 2 (congruency; congruent, incongruent) × 2 (proportion congruent; high [75% congruent/25% incongruent], low [25% congruent/75% incongruent]), repeated-measures ANOVA was performed on these data. The analysis revealed a main effect of congruency, *F*(1, 42) = 81.55, *p* ≤ .0001, η_p_^2^ = .66, with the expected higher sensitivity for congruent trials than incongruent trials, suggesting a failure to selectively attend to the task-relevant part of the lines. There was no main effect of proportion congruent, *F*(1, 42) = 0.15, *p* = .70, η_p_^2^ = .0036. However, there was a significant interaction between congruency and proportion congruent, *F*(1, 42) = 7.64, p = .0084, η_p_^2^ = .15, with a larger effect of congruency in the high, relative to the low, proportion congruent condition (see Fig. 4A.). The impact on the congruency effect, an index of holistic processing, of manipulating the probability that the task-relevant part will contain congruent information is broadly consistent with learned attentional accounts of holistic processing. That is, the degree to which the face stimuli were processing holistically was shaped by information learned via experience with the task.

#### RT

The mean RT for each condition for correct trials only is presented in Fig. 4B. A 2 (congruency; congruent, incongruent) × 2 (proportion congruent; high [75% congruent/25% incongruent], low [25% congruent/75% incongruent]), repeated-measures ANOVA was performed on these data. The analysis revealed a main effect of congruency, *F*(1, 42) = 25.03, *p* ≤ .0001, η_p_^2^ = .37, with the expected faster RTs for congruent trials than incongruent trials. There was no main effect of proportion congruent, *F*(1, 42) = 0.74, *p* = .39, η_p_^2^ = .017, or interaction between congruency and proportion congruent, *F*(1, 42) = 2.16, p = .15, η_p_^2^ = .049 (see Fig. 4.).

#### Comparison between faces and gestalt stimuli

To statistically compare the effects found for the Gestalt stimuli to those found for faces in Experiment 1, a 2 (stimulus type; faces, Gestalt line patterns) × 2 (congruency; congruent, incongruent) × 2 (proportion congruent; high [75% congruent/25% incongruent], low [25% congruent/75% incongruent]), mixed repeated-measures ANOVA was performed on these data. This analysis was not included in the preregistration. This analysis of the sensitivity (d’) data revealed a main effect of stimulus type, *F*(1, 83) = 16.19, *p* ≤ .0001, η_p_^2^ = .16, with higher sensitivity for trials with the line patterns than for those with faces. However, there were no interactions between stimulus type and the other variables in the analysis (all ps > .13). Unsurprisingly, and consistent with the separate analyses of the data from Experiments 1 and 2, there was a significant interaction between congruency and proportion congruent, *F*(1, 83) = 12.56, p = .00807, η_p_^2^ = .13, with a larger effect of congruency in the high, relative to the low, proportion congruent condition. There was also the expected main effect of congruency, *F*(1, 83) = 43.56, *p* ≤ .0001, η_p_^2^ = .70, with the expected higher sensitivity for congruent trials than incongruent trials, but no main effect of proportion congruent, *F*(1, 83) = 0.32, *p* = .57, η_p_^2^ = .0039.

The results of the same analysis performed on the RT data were also consistent with those from the separate analyses of the data from Experiments 1 and 2, revealing only a main effect of congruency, *F*(1, 83) = 53.08, *p* ≤ .0001, η_p_^2^ = .39, with the expected faster RTs for congruent trials than incongruent trials, but no main effect of proportion congruent, *F*(1, 83) = 0.30, *p* = .59, η_p_^2^ = .0036. There was no main effect of stimulus type, *F*(1, 83) = 0.14, *p* = .70, η_p_^2^ = .0017, or interactions between any variables in the analysis (all ps > .25). Therefore, the impact of manipulating the probability that the task-relevant part will contain congruent information on the congruency effect, an index of holistic processing, was statistically indistinguishable for faces and the Gestalt line pattern stimuli.

## General discussion

The findings reported here are consistent with the hypothesis that holistic processing is affected by learned patterns of attentional allocation both for faces (Experiment 1) and for non-face stimuli (Experiment 2). For both stimulus types, the probability that the task-irrelevant (uncued) part would contain congruent (or alternatively incongruent) information impacted the degree to which that part influenced task performance. Notably, the typical inability of participants to make a judgement about part of an object without interference from another, task-irrelevant, part is the basis of a key hallmark of holistic processing. Thus, these results demonstrate that participants can modulate the degree to which they process face and non-face stimuli holistically depending on the likelihood that this strategy would lead them to process information that could support or interfere with their performance.

The findings reported here are in line with previous work demonstrating that learned attention to parts of stimuli can shape the degree to which both face and non-face stimuli are processed holistically (e.g., Chua et al., 2014, 2015). For example, training on a task where a specific part is consistently not relevant for distinguishing different exemplars within non-face and face (novel race) stimuli results in reduced holistic processing of these stimuli, compared to a training history where both parts were diagnostic (Chua et al., 2014, 2015). Notably, in contrast with previous studies, in the current study there was no consistency or predictability with respect to which part or spatial region would contain task-relevant information. Instead, the task context was such that it either did or did not encourage holistic processing of the faces. Due to this, effects of holistic processing could not be accounted for by suggestions that holistic processing emerges largely from learned prioritisation of specific parts or stimulus regions (e.g., Chua et al., 2015). Thus, the current findings extend the role of learned attention patterns to include cases where attention is shaped by the probability that non-cued parts would contain congruent or incongruent information.

Notably, in the context of the standard composite part matching task, processing the stimulus holistically has always been detrimental to performance in the incongruent condition. However, the benefits of processing the stimulus holistically in the congruent condition likely compensate for this cost, undermining any potential motivation to reduce the degree to which the stimuli are processed holistically. Thus, in the typical composite task, there is little strategic benefit to engaging in the cognitively effortful process of trying to override a learned or default tendency to attend to the stimulus as a whole.

Previous studies have demonstrated that holistic processing still occurs even when there is no uncertainty about which part will be task-relevant (e.g., when participants always make the part judgements about one half of a face or object of expertise throughout the entire task (Curby & Gauthier, 2014; Gauthier et al., 2003). Although, once again, in this context the benefits to performance in the congruent condition likely compensate for this cost, undermining motivation to reduce holistic processing and thus to actively ignore the part that was always task-irrelevant. The findings of the current study suggest that it is only when the task context is such that these costs and benefits are uneven, as in the case of the high and low congruency probability conditions we used in the current study, that people change how they processed the face parts.

While our findings are inconsistent with traditional template-based accounts of holistic processing, it is possible that impacts of attentional manipulations on holistic processing operate via impacting the degree to which a stimulus matches an internal face template. Although it is unclear how such an account could explain the mirroring effects found for the face and non-face stimuli as there is no reason to believe that participants would have an internal template of the novel non-face stimuli used in Experiment 2. More generally, the degree to which early perceptual processes are penetrable by attentional manipulations is related to a topic of a lively ongoing debate (e.g., see Firestone & Scholl, 2016). On a related note, the degree to which learned attention may impact early perceptual processing, including that supporting holistic processing, is a worthy topic for future research.

One possibility is that the congruency probability manipulation impacted holistic processing by affecting the degree to which an object-level representation of a stimulus influenced or guided attention, that is, the degree to which attention was object-based. Nah and Shomstein (2020) demonstrated that although spatial attentional biases are prioritised over contributions from more object-level attentional biases, both guide attention to and within objects. Previous research has also demonstrated that attentional mechanisms, such as those supporting object-based attention, are sensitive to the probability that particular regions within, or across, objects will contain task-relevant information (Shomstein & Yantis, 2004). For example, while observers are faster to attend to regions within a cued object by default, compared to equidistant regions within another object (Egly et al., 1994), manipulations of the probability of where task-relevant information will appear can override this default bias (Shomstein & Yantis, 2004). Similarly, here we show that while we tend to processes faces holistically (attending to both the top and bottom parts of the face), this attentional strategy can be attenuated by probabilistic manipulations of the likelihood that the other, non-target, region will contain potentially helpful (congruent) or misleading (incongruent) information.

Previous studies have demonstrated that the impact of a distractor stimulus is reduced when participants learned implicitly about the probability that a distractor was present, regardless of whether they were explicitly aware of where the distractor was likely to appear (Gao & Theeuwes, 2022; Gaspelin & Luck, 2018; Luck et al., 2021). Consistent with this suggestion, informing participants in advance about the likely presence and location of a salient distractor stimulus does not help them protect their performance from its effects (Wang & Theeuwes, 2018a). However, implicitly learning the probability of the location of a distractor stimulus appears to elicit suppression of that location (Wang & Theeuwes, 2018a, 2018b). Notably, in the current study participants did not report being aware of the congruency probability manipulation when asked about the difference between the two sessions, which suggests that the impact of the manipulation on their performance was implicit, not unlike that which appears to underlie the reduction in attentional capture by distractor stimuli. This shared characteristic with established attentional effects provides general support for an attention-based account of our findings and suggest that future studies should explore the possibility that there is a suppression of the task-irrelevant part in the low-congruency condition.

It is unclear from the current study if the observed effect is driven by an increase in holistic processing in the high congruent probability condition, a decrease in holistic processing in the low congruent probability condition, or a combination of both. For example, in the high-congruency condition there might be increased allocation of attention to the task-irrelevant part because it is often compatible with the correct response. Alternatively, participants might reduce their effort to limit their attention to the task-relevant part, or suppress the task-irrelevant part, given that they can still achieve a high level of performance in the high-congruency condition even when they don’t restrict their attention. These effects may be akin to probability cueing effects established in the attention literature (e.g., probability cueing effects for distractor locations; Goschy et al., 2014). Prior probability cueing research suggests that there are likely contributions from both a prioritisation of the congruent task-irrelevant part and a modification of the degree to which the task-irrelevant part is suppressed (Ferrante et al., 2018). Future studies are needed to better elucidate what changes, in terms of how the task-relevant and/or task-irrelevant parts are processed, drive the effects reported here.

Another possibility is that the modulation of holistic processing by the probability manipulation may reflect a strategic narrowing of attention when viewing the second stimulus, while a high congruent task context may encourage the relaxing of attentional control, broadening the focus of attention. Previous studies have demonstrated effects on holistic processing akin to this as a result of task contexts that encourage, or prime, a more global versus local processing strategy (e.g., Gao et al., 2011). However, the manipulations used in previous studies, unlike that in the current study, were external to the face task, and thus their impact did not reflect a strategic shaping of the processing of the target stimulus, but potentially more of a processing carry-over effect.

It is worth noting that the probability effect demonstrated here was based on exposure to the task-context over only six blocks of 64 trials. Given our exposure to stimuli in the real-world is many-fold this, such probability manipulations have the potential to be a powerful force in shaping holistic processing. This might be especially the case in the context of perceptual expertise, where experts often accumulate years of experience with specific stimuli and tasks. A lengthier training or task context period, during which the effect could potentially grow, may have been able to produce a stronger effect. It is also an open question as to whether the effect would be stronger if participants were explicitly told of the different congruency probabilities across the two sessions. However, it is possible that this effect is established only through direct experience processing the stimuli in this context, and thus there may be no additional benefit of explicitly informing participants about the probability manipulation. Future research should explore this possibility.

An open question arising from the findings reported here is the degree to which other measures or indices of holistic processing, such as the inversion effect or part-whole effect, would be similarly impacted by probabilistic-based attentional manipulations like that used in the current study. There is consensus within the literature that holistic processing is unlikely to be a unitary construct, but instead may have several distinct components (Boutet et al., 2021). Even within attentional accounts there are potentially distinct mechanisms supporting holistic processing. Specifically, we have proposed previously that holistic processing can arise either from a learned attentional strategy, in the case of objects of expertise (including both face and non-face objects), or an attentional approach that is afforded by the nature of the stimuli (i.e., the presence of strong Gestalt cues such as those in faces and the non-face line stimuli used in Experiment 2) or both (Curby et al., 2019). Thus, while it is beyond the scope of this study to speak to the degree to which different measures of holistic processing would be impacted by probabilistic (attentional) manipulations like that used here, this would be a worthwhile question for further research.

In the real world the location of diagnostic information for many tasks is not absolute and instead follows a probability distribution. For example, the location of a chest nodule in a radiograph follows a specific probability distribution, with chest nodules being most likely present in the upper right quadrant of the chest (e.g., Garland, 1961). Thus, there is frequently statistical information available from experience that experts can use to shape how they attend to a stimulus (e.g., see Carrigan et al., 2019, for a study exploring this possibility in radiologists). In this context, the development of a holistic processing strategy may result, in part, from the task demands of identifying stimuli where spatially distributed regions share a similarly high probability of containing task-relevant information. However, holistic processing of novel stimuli, with which participants have had no chance to establish prior probabilities of the expected location of diagnostic information, suggests that other factors such as the presence of strong Gestalt grouping cues, and thus potentially strong object-based attention, may also drive a distributed default allocation of attention. Nonetheless, here we show that observers can adjust the degree to which they adopt a more holistic processing strategy depending on the degree to which this strategy is suitable given the current task context.

In summary, this study provides evidence that holistic processing can be shaped by the task context, or more specifically the experienced probability that the task-irrelevant part of a stimulus will contain congruent or incongruent information. While broadly consistent with a role of learned attention in driving holistic processing, this effect extends previous findings suggesting that holistic processing is a result of learned prioritisation of specific regions or parts of stimuli. Instead, our findings suggest that task history can also modulate holistic processing via mechanisms that do not rely upon consistency in the spatial location of diagnostic information across trials, but that are shaped more broadly by the likely consequences of adopting a holistic strategy. Given the established link between holistic processing and visual expertise, these findings further illuminate the complex mechanisms underlying skilled visual performance.

## Footnotes

1 The pre-registered sample size was a minimum of 40 with the goal of 46.

2 Some versions of the composite task also include a misaligned control condition. Our design only contained the necessary conditions to test our hypothesis as including an additional condition such as this would have required us to move to a between-subjects design (due to the length of the study, i.e., 4 × 45 min sessions) and rendered us unable to control for individual differences in holistic processing.

3 The pre-registered sample size was a minimum of 40 with a goal of 46. Forty-nine participants were recruited as we slightly overcompensated for the tendency of some participants to fail to attend the lab and complete the study after signing up online.
